# Intracellular pHluorin as Sensor for Easy Assessment of Bacteriocin-Induced Membrane-Damage in *Listeria monocytogenes*

**DOI:** 10.3389/fmicb.2018.03038

**Published:** 2018-12-11

**Authors:** Peter Crauwels, Leonie Schäfer, Dominik Weixler, Nadav S. Bar, Dzung B. Diep, Christian U. Riedel, Gerd M. Seibold

**Affiliations:** ^1^Institute of Microbiology and Biotechnology, University of Ulm, Ulm, Germany; ^2^Department of Chemical Engineering, Norwegian University of Science and Technology (NTNU), Trondheim, Norway; ^3^Faculty of Chemistry, Biotechnology and Food Science, Norwegian University of Life Sciences, Ås, Norway

**Keywords:** Listeria, pH sensor, membrane damage, bacteriocins, fluorescence

## Abstract

Bacteriocins are antimicrobial peptides naturally produced by many bacteria and were shown to be effective against various pathogens including *Listeria monocytogenes*. *L. monocytogenes* is a food-borne pathogen that frequently causes disease outbreaks around the world with fatal outcomes in at-risk individuals. Thus, bacteriocins are a promising solution to prevent contaminations with *L. monocytogenes* and other microorganisms during food production and preservation. In the present study, we constructed *L. monocytogenes* EGD-e/pNZ-P_help_-pHluorin, a strain that constitutively expresses the pH-sensitive fluorescent protein pHluorin, as a sensor strain to detect disruption of the pH gradient by the membrane-damaging activity of bacteriocins. The ratiometric fluorescence properties of pHluorin were validated both in crude extracts and permeabilized cells of this sensor strain. *L. monocytogenes* EGD-e/pNZ-P_help_-pHluorin was used to assess membrane damaging activity of the bacteriocins nisin A and pediocin PA-1 and to determine the minimal concentrations required for full disruption of the pH gradient across the membrane. Moreover, the sensor strain proved useful to analyze the presence of compounds affecting membrane integrity in supernatants of a nisin Z-producing *Lactococcus lactis* strain at different timepoints during growth. Supernatants of this strain that were active in disrupting the pH gradient across the membrane were also shown to inhibit growth of *L. monocytogenes*. In summary, the presented results suggest that the generated sensor strain is a convenient, fast and reliable tool to identify and characterize novel bacteriocins and other compounds that target membrane integrity.

## Introduction

*Listeria monocytogenes* is a saprophytic soil organism found in a wide range of habitats as well as a food-spoiling bacterium with the potential to cause life-threatening disease in humans (Vivant et al., 2013; Ferreira et al., 2014). In healthy individuals, food-borne infections with *L. monocytogenes* result in mild gastroenteritis or remain completely asymptomatic. However, in at-risk groups such as immunocompromised persons, elderly people, newborns and pregnant women, *L. monocytogenes* may cause severe to fatal disease (Vázquez-Boland et al., 2001; Allerberger and Wagner, 2010). Its extremely high tolerance to a wide range of environmental conditions and stresses and the ability to form biofilms on abiotic surfaces make *L. monocytogenes* a major concern in food processing and sanitation of the respective production lines (Ferreira et al., 2014; NicAogáin and O’Byrne, 2016).

Despite an increased risk of bacterial contamination with human pathogens such as *L. monocytogenes* and the associated, far reaching consequences for safety, health, environment and profitability of consumers and producers, the interest in minimally processed foods is steadily increasing. This poses the problem of reducing the risk for contamination with pathogens and food-spoiling microorganisms during production, processing and storage without affecting taste, texture and quality. One possibility to address these challenges that has gained increasing interest in recent years is the use of naturally occurring antimicrobial peptides (AMPs) such as bacteriocins, which are produced by a wide range of bacteria (Cotter et al., 2013; Silva et al., 2018). Bacteriocins are small heat-stable antimicrobial peptides of (usually) 30–60 amino acid residues (Cotter et al., 2013; Chikindas et al., 2018) and are able to suppress growth and/or directly kill target organisms. However, current approaches to identify, purify and characterize bacteriocins are limited to time- and labor-intensive genome mining and growth-dependent assays (Sandiford, 2017; Zou et al., 2018). Also, these approaches do not provide mechanistic information as to whether a bacteriocin is biostatic, biocidal, or bacteriolytic and whether it is able to cause membrane damage or pore formation.

Bacteriocins can affect the integrity of the cell envelope and/or interfere with cell metabolism (Cotter et al., 2013). For example, nisin acts by distinct mechanisms (Bierbaum and Sahl, 2009). Binding of nisin to its receptor lipid II, an important intermediate of peptidoglycan, inhibits growth by blocking cell wall biosynthesis. At the same time, nisin and lipid II form complexes that destabilize the plasma membrane and from pores. At higher concentrations, nisin also forms pores in the absence of lipid II as shown by model lipid layers. The receptors for pediocins and other class II bacteriocins are surface proteins including sugar and amino acid transporters (Gabrielsen et al., 2012; Oppegård et al., 2016; Tymoszewska et al., 2017; Ríos Colombo et al., 2018), the stress response protease RseP (Ovchinnikov et al., 2017), or enzymes of cell wall synthesis (Kjos et al., 2014).

*Listeria monocytogenes* is able to grow over a wide range of pH from 4.3 to 9.4 (te Giffel and Zwietering, 1999; Cheng et al., 2015). This is of particular importance because this organism has to cope with a wide range of habitats with extreme pH values including soil, acidic food matrices, the stomach and lower intestine, and the phagosome of macrophages and other host cells (Cotter et al., 2001; Gray et al., 2006; Bierne et al., 2018). Despite exposure to extreme external pH (pH_o_), *L. monocytogenes* is able to maintain an intracellular pH (pH_i_) within a narrow range of 7.6–8.0 (Shabala et al., 2002a,b; Cheng et al., 2015) by efficient mechanisms for pH-homeostasis (Gray et al., 2006). Together with the electrical membrane potential ΔΨ, the pH gradient across the membrane constitutes the proton motive force, which is essential for generation of ATP by the F_1_F_0_ATPase (Booth, 1985; Cotter et al., 2000). Failure to maintain pH homeostasis, e.g., following exposure to bacteriocins such as pediocin and nisin, leads to loss of cell viability and, therefore, may be used as a sensitive indicator of bacterial death at the single cell level (Budde and Jakobsen, 2000; Kastbjerg et al., 2009).

For the determination of pH_i_ in bacteria and other cells, ratiometric fluorescent proteins such as the pH-sensitive green fluorescent protein pHluorin (Miesenböck et al., 1998), its derivative pHluorin2 (Mahon, 2011), and the red fluorescent protein pHuji (Shen et al., 2014) have been developed and applied. pHluorin and pHluorin2 produce two excitation peaks: one that increases in intensity with increasing pH and one that decreases with increasing pH. Compared to fluorescent or radioactive dyes, which are also used for determination of pH_i_ (Kashket, 1985; Han and Burgess, 2010), the use of ratiometric fluorescent proteins offers several advantages. An organism of choice can be genetically modified to readily express these proteins. This prevents the need of adding a compound exogenously and allows rapid and non-invasive measurements of pH_i_ online during cultivation of bacteria (Martinez et al., 2012; van Beilen and Brul, 2013).

The aim of the presented study was to establish a simple, fast, reliable and cost-effective assay to assess membrane damage of in *L. monocytogenes* using pHluorin.

## Materials and Methods

### Bacterial Strains and Growth Conditions

All strains used in this study are listed in Table 1. The serotype 1/2a strain *L. monocytogenes* EGD-e (Bécavin et al., 2014) was used to generate the sensor strain for detection of membrane damage. *E. coli* DH10B (Invitrogen, Darmstadt, Germany) was used as a cloning host. *L. lactis* strain B1627 (strain collection Dzung B. Diep, NMBU, Norway) was used to produce nisin-containing culture supernatants and *L. lactis* MG1363 (Gasson, 1983) served as nisin-negative control. Bacteria were grown under standard conditions in LB (*E. coli*), BHI (*L. monocytogenes*) or GM17 (*L. lactis*) broth aerobically at 37°C with agitation (*E. coli*, *L. monocytogenes*) or 30°C without aeration (*L. lactis*; Kuipers et al., 1995). Where indicated chloramphenicol was added to the media at a final concentration of 20 μg/ml (*E. coli*) or 15 μg/ml (*L. monocytogenes*).

**Table 1:** Bacterial strains, plasmids and oligonucleotides used in this study.

Strain	Relevant characteristics	Source
*E. coli*		
DH10B	cloning host	Invitrogen^TM^
*Listeria monocytogenes*		
EGD-e	type strain, serotype 1/2a	Bécavin et al., 2014
EGD-e/pNZ44	empty vector control strain	this study
EGD-e/pNZ-P_help_-pHluorin	strain with high level, constitutive expression of pHluorinLmo	this study
*L. lactis*		
MG1363	intestinal isolate from a breast-fed infant	Gasson, 1983
B1627	natural isolate producing nisin Z	strain collection D. Diep, NMBU

**Plasmid**	**Relevant characteristics**	**Source**

pNZ44	shuttle vector, replicon for *E. coli* and a wide range of Gram-positive bacteria, high-copy number, Cm^R^	McGrath et al., 2001
pIMK2	?Site-specific listerial integrative vector for constitutive overexpression, Kan^R^	Monk et al., 2008
pNZ-P_help_-pHluorin	derivative of pNZ44, plasmid for high level, constitutive expression of pHluorinLmo	this study

**Primer**	**Sequence (5′ 3′)**	**Amplicon [bp]**

P35	TTTTTATATTACAGCTCCAAGACGTCGGACCTTTCGTTTT	1205
P36	AGTTCTTCTCCTTTCGCCATGGGTTTCACTCTCCT	
P37	AGGAGAGTGAAACCCATGGCGAAAGGAGAAGAACT	779
P38	AGTGGTACCGCATGCCTGCATACCTGGGATCCGTCGACCT	

**Gene**	**Sequence (5′ 3′)**	**Size [bp]**

pHluorinLmo	ATGGCGAAAGGAGAAGAACTATTTACTGGCGTTGTACCGATTTTAGTGGA	717
	ATTAGATGGCGATGTAAATGGCCATAAGTTTAGTGTTTCTGGTGAAGGTGA	
	AGGGGATGCAACCTATGGCAAATTAACGCTTAAATTCATCTGTACAACTGG	
	GAAATTACCAGTTCCTTGGCCTACACTAGTAACTACCTTTTCCTATGGTGTT	
	CAATGCTTTAGTCGTTATCCAGACCACATGAAACGTCATGACTTCTTTAAAT	
	CTGCAATGCCAGAAGGCTATGTTCAAGAACGAACAATCTTCTTTAAAGATG	
	ACGGTAACTATAAAACGAGAGCTGAAGTGAAATTTGAAGGAGATACGTTA	
	GTCAATCGCATTGAATTGAAAGGGATAGATTTCAAAGATGACGGAAACATTT	
	TAGGTCATAAACTTGAATACAACTATAATGAGCATCTAGTATACATAATGGCTG	
	ATAAGCAAAAGAATGGTACAAAAGCGATCTTTCAAGTGCATCACAACATTGA	
	AGATGGAGGTGTTCAATTAGCCGATCACTATCAACAGAATACACCAATTGGA	
	GATGGACCAGTGTTATTACCAGACAATCACTACCTTCATACACAATCAGCAC	
	TTAGCAAAGATCCGAATGAGAAACGTGATCATATGGTCTTGTTAGAGTTTGTA	
	ACAGCTGCGGGAATTACTCATGGTATGGATGAATTGTACAAATAA	

For preparation of supernatants, overnight (o/N) cultures of *L. lactis* strains were diluted in 20 ml of fresh GM17 broth in an Erlenmeyer flask to an optical density at 600 nm (OD_600_) of 0.1 and grown under standard conditions. Growth was monitored by measuring OD_600_. At the indicated timepoints, aliquots were collected and supernatants were prepared by centrifugation at 3200 × *g* for 3 min and filtration (pore size: 0.2 μm).

### Cloning Procedures

The gene for ratiometric pHluorin (Miesenböck et al., 1998) was cloned under control of the strong constitutive P_help_ promoter (Riedel et al., 2007) into the back-bone of the *E. coli*/Gram-positive shuttle vector pNZ44 (McGrath et al., 2001). A pHluorin coding sequence, which was codon-optimized for *L. monocytogenes* (*pHluorinLmo*; Table 1) was synthesized and purchased as 752 bp DNA fragment from a commercial service provider (Eurofins Genomics, Ebersberg, Germany) and cloned into pJET1.2 according to manufacturer’s instructions (Thermo Scientific, Waltham, MA, United States). The *pHluorinLmo* gene and a DNA fragment comprising the *rrnB* T1 transcription terminator and the P_help_ promoter of pIMK2 (Monk et al., 2008) were amplified by PCR using Phusion High-Fidelity DNA Polymerase (ThermoFisher Scientific, Waltham, MA, United States) and primer pairs P35/P36 and P37/P38, respectively. All primers are listed in Table 1 and were designed to contain overlapping homologous sequences for either the plasmid pNZ44 (P35 and P38) or the respective other PCR fragment (P36 and P37). The vector backbone of pNZ44 (McGrath et al., 2001) was linearized by restriction digest using BglII and PstI and gel purified thereby removing the *p44* promoter. The pNZ44 back-bone and both PCR fragments were fused in a single reaction by Gibson Assembly (Gibson et al., 2009) and the resulting plasmid pNZ-P_help_-pHluorin was transformed into chemically competent *E. coli* DH10B using standard protocols. Plasmids of positive clones were isolated and, following verification by restriction analysis and sequencing, transformed into *L. monocytogenes* EGD-e using a previously described protocol (Monk et al., 2008).

### Preparation of Crude Extracts

To analyze the pH-dependent ratiometric change in fluorescence properties of pHluorin, crude extracts of *L. monocytogenes* EGD-e/pNZ-P_help_-pHluorin and *L. monocytogenes* EGD-e/pNZ44 were prepared. For this purpose, an o/N culture was diluted in 20 ml of sterile BHI broth to an OD_600_ of 0.1 and incubated for 3–4 h at 37°C with agitation. A 10 ml aliquot of the culture was centrifuged (3200 × *g*, 10 min, 4°C). The pellet was washed once with PBS, resuspended in 0.5 ml in 1M K_2_HPO_4_ in H_2_O, and transferred to a 1 ml cryotube containing 250 μg silica beads (diameter 1 mm, Sigma-Aldrich, Taufkirchen, Germany). Cells were disrupted by 3 cycles of 30 s each at 6500 rpm in a Precellys^®^ homogenizer (PEQLAB, Erlangen, Germany) with cooling on ice between cycles. Subsequently, silica beads and cell debris was precipitated by centrifugation for 30 min at 14000 × *g* and 4°C and supernatants, i.e., the crude extracts, were stored at -20°C until further use.

One aliquot of each crude extract was subjected to heat inactivation for 10 min at 90°C. The protein content of all extracts was determined by BCA assay (ThermoFisher Scientific, Waltham, MA, United States) and equal amounts of protein of each extract were analyzed by SDS polyacrylamide gel electrophoresis on a 10% acrylamide gel in a TRIS/glycine buffer system. Following separation, gels were imaged on a iBright^TM^ FL1000 imaging system (Invitrogen, Darmstadt, Germany) to visualize pHluorin (excitation at 455–485 nm; emission at 510–555 nm) and the PageRuler^TM^ Prestained Protein ladder (ThermoFisher Scientific, Waltham, MA, United States; excitation at 610–635 nm; emission at 675–720 nm).

### Fluorescence Measurements

To analyze fluorescence properties of pHluorin, 400 μl of crude extract were mixed with 600 μl of 1M K_2_HPO_4_ in H_2_O and transferred to a quartz cuvette equipped with magnetic stirrer and pH electrode. In this setting, crude extracts had an initial pH of 8.6. pH were titrated with 1M HCl to the indicated values. At each pH, an excitation scan at 350–490 nm excitation (emission at 510 nm) was performed using a Aminco Bowmann^®^ Series 2 Luminescence Spectrometer (SLM Instruments, Urbana, IL, United States) with a scan rate of 8 nm/s.

Fluorescence measurement using *L. monocytogenes* cells were performed in Listeria minimal buffer (LMB), which is based a modified synthetic minimal medium for *L. monocytogenes* (Tsai and Hodgson, 2003) and is composed as follows: 100 mM MOPS, 4,82 mM KH_2_PO_4_, 11.55 Na_2_HPO_4_, 1.7 mM MgSO_4_, 0.6 mg/ml (NH_4_)_2_SO_4_, 55 mM glucose, pH 6.5. For measurements, *L. monocytogenes* EGD-e/pNZ-P_help_-pHluorin was grown in BHI over night (typically 16h), washed once in PBS and adjusted to an OD_600_ of 3 in filter-sterilized (pore-size 0.2 μm) LMB. Aliquots of 100 μl were distributed into single wells of a black microtiter plate and mixed with 100 μl of LMB containing nisin A or pediocin PA-1 at the indicated concentrations, sterile GM17 medium, or supernatants of *L. lactis* strains grown in GM17. Following an incubation of 1h at room temperature, fluorescence at 510 nm (emission) was measured after excitation either by scanning a range of wave lengths (350–490 nm) or at the excitation maxima of pHluorin (400 and 470 nm) using a Tecan Infinite^®^ M200 multimode plate reader (Tecan, Crailsheim, Germany).

### Bacteriocins

A nisin A stock solution was prepared using a commercial nisin preparation containing 2.5% of active compound (Sigma-Aldrich, Taufkirchen, Germany; 1 009 000 IU/g). The stock solution contained 25 mg/ml of active nisin A, i.e., a potency of 25 225 IU/ml. Pediocin PA-1 was purchased as a stock solution of 0.1 mg/mL (22 nM) in 0.1 M sodium acetate pH 5.0 (Sigma-Aldrich, Taufkirchen, Germany). Nisin A and pediocin PA-1 stock solutions were diluted in LMB to yield final concentrations in the assay as indicated in the figures.

### Microscopy – Live Dead Staining

A fresh o/N culture of *L. monocytogenes* EGD-e washed one in PBS and bacteria were resuspended in LMB at OD_600_ = 3. Aliquots of 100 μl were mixed with a cetyltrimethylammonium bromide (CTAB) stock solution to give final CTAB concentrations of 0, 0.001, 0.005, and 0.01 % (w/v) and incubated for 1h in the dark. Then, bacteria were stained using the LIVE/DEAD BacLight Bacterial Viability Kit (L7012, Invitrogen, Darmstadt, Germany). The dyes of this kit are Syto^®^ 9 and propidium iodide staining all or only dead cells with a compromised membrane, respectively. Following staining, samples were washed and imaged using a Axio Observer Z1 (Zeiss, Oberkochen, Germany) using filters for Syto^TM^ 9 (excitation at 450–490 nm; emission at 515–565 nm) and propidium iodide (excitation at 575–625 nm; emission at 660–710 nm). Images were acquired with a 100× objective and analyzed using the Zen software (Version 2.3 SP1; Zeiss).

### Growth Inhibition of *L. monocytogenes* by *L. lactis* Supernatants

The effect of *L. lactis* supernatants on growth of *L. monocytogenes* EGD-e/pNZ-P_help_-pHluorin was assessed by diluting an o/N culture in fresh BHI broth to an OD_600_ of 0.1 and then mix at a 1:1 ratio with either GM17 (positive control), GM17 containing 0.01% (w/v) CTAB (negative control, final CTAB concentration 0.005%) or supernatants of *L. lactis* cultures. Aliquots of 200 μl of these mixes were distributed in technical duplicates in individual wells of transparent, flat-bottom 96-well plates (Sarstedt, Nümbrecht, Germany) and incubated with gentle shaking at 37°C. Growth was monitored by measuring OD_600_ in a Tecan Infinite M200 multimode plate reader (Tecan, Crailsheim, Germany).

### Statistical Analysis

Data was analyzed by one-way ANOVA using GraphPad Prism 6 (GraphPad Software, La Jolla, CA, United States). Dunnett’s post-test was used to calculate *p*-values adjusted for multiple comparisons with the negative control set as the reference condition. Differences were considered statistically significant at *p* < 0.05.

## Results

**FIGURE 1:**
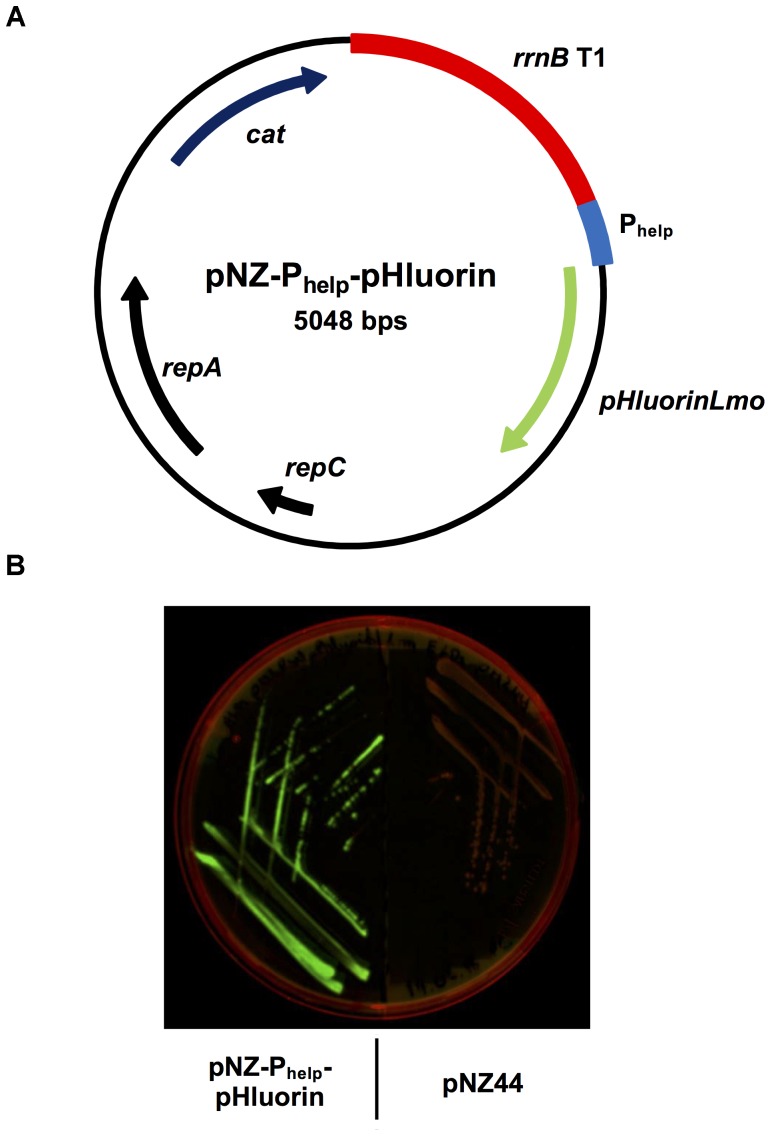
Generation of the sensor strain *L. monocytogenes* EGD-e/pNZ-P_help_-pHluorin expressing ratiometric pHluorin. **(A)** Plasmid map of pNZ-P_help_-pHluorin for high level, constitutive expression of pHluorin encoded by the codon-optimized *pHluorinLmo* gene. **(B)** *L. monocytogenes* EGD-e/pNZ-P_help_-pHluorin (left) and *L. monocytogenes* EGD-e/pNZ44 (right) on BHI agar plate containing 15 μg/ml chloramphenicol. Expression of pHluorin was visualized with excitation at 455–485 nm and emission at 510–555 nm.

In order to generate a sensor strain for the detection of membrane damage, a plasmid was constructed based on the broad host-range vector pNZ44 (McGrath et al., 2001) containing the gene for the pH-sensitive protein ratiometric pHluorin (Miesenböck et al., 1998) codon-optimized for *L. monocytogenes* (*pHluorinLmo*; Table 1) under the control of the strong, constitutive, synthetic promoter P_help_ (Riedel et al., 2007). In brief, two overlapping fragments, one containing the P_help_ sequence and the other containing the *pHluorinLmo* sequence, were generated by PCR. Using Gibson assembly (Gibson et al., 2009), both fragments were assembled into the pNZ44 backbone, resulting in the plasmid pNZ-P_help_-pHluorin (Figure 1A). Following transformation of this plasmid into *L. monocytogenes* EGD-e, the recombinant strain *L. monocytogenes* EGD-e/pNZ-P_help_-pHluorin could be easily distinguished by bright green fluorescence from the isogenic strain carrying the empty vector pNZ44 on agar plates imaged following excitation at 455–485 nm (Figure 1B).

**FIGURE 2:**
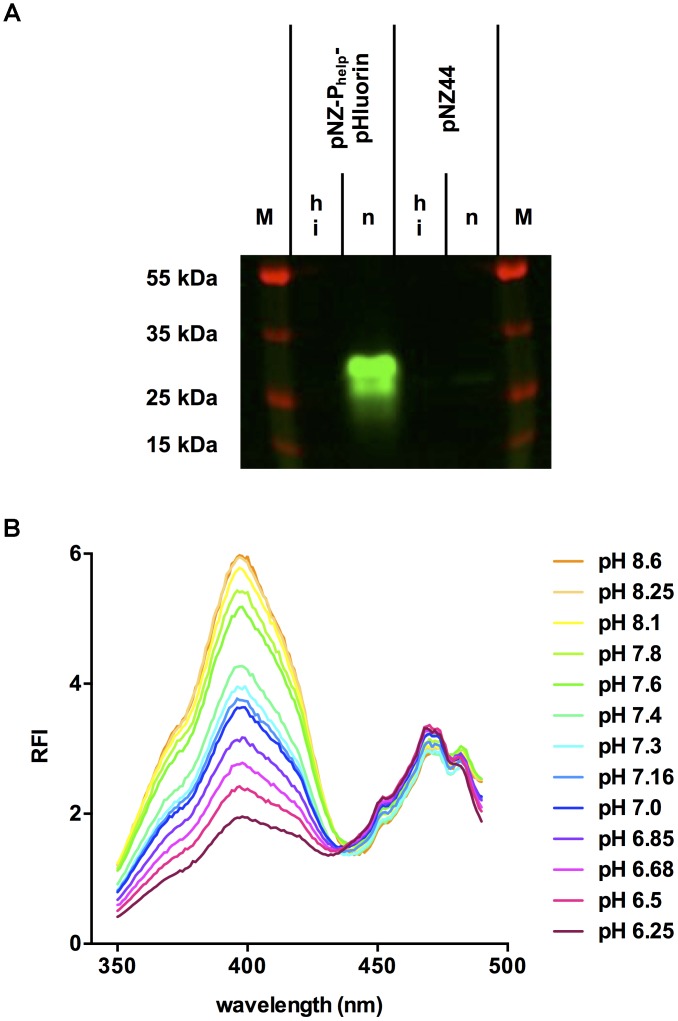
Fluorescence properties of pHluorin in crude extracts of *L. monocytogenes* EGD-e/pNZ-P_help_-pHluorin. **(A)** Native (n) or heat-inactivated (hi) crude extracts of *L. monocytogenes* EGD-e/pNZ-P_help_-pHluorin and *L. monocytogenes* EGD-e/pNZ44 were resolved by SDS-PAGE. The image of the gel is an overlay of the channels for pHluorin (excitation at 455–485 nm; emission at 510–555 nm) and protein marker (excitation at 610–635; emission at 675–720 nm). **(B)** Crude extracts were diluted in LMB and pH was adjusted with 1M HCl. At the indicated pH values, excitation spectra (350–490 nm) were aquired measuring relative fluorescence intensity (RFI) at 510 nm emission.

Crude extracts of *L. monocytogenes* EGD-e/pNZ-P_help_-pHluorin contained a single fluorescent protein with the predicted size of pHluorin (approx. 27 kDa; Figure 2A). This protein was absent in crude extracts of the vector control strain and lost its fluorescent properties upon heat denaturation. Fluorescent excitation spectra of crude extracts in buffer at different pH revealed a change in emission at 510 nm characteristic for pHluorin (Figure 2B) and a ratiometric increase in the emission peak at around 400 nm excitation with decreasing pH.

**FIGURE 3:**
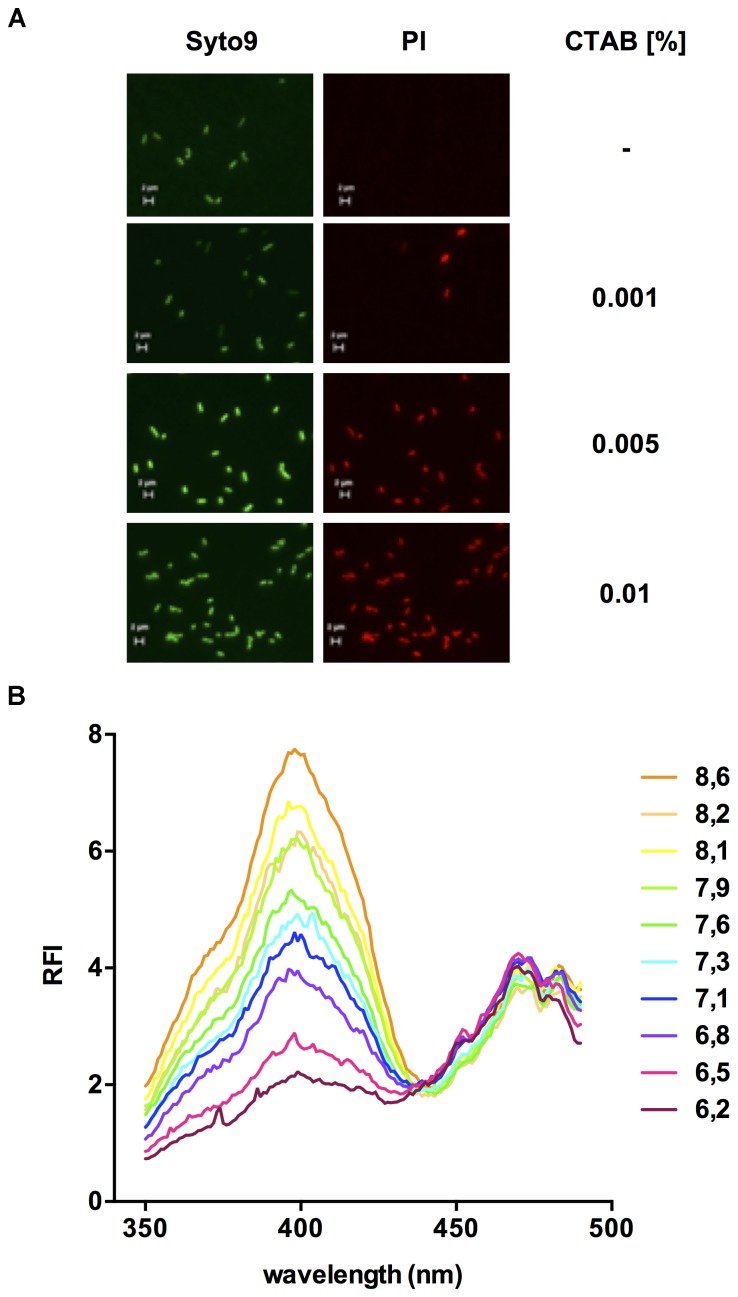
Fluorescence properties of permeabilized *L. monocytogenes* EGD-e/pNZ-P_help_-pHluorin bacteria. **(A)** In order to determine the appropriate concentration for permeabilization, *L. monocytogenes* EGD-e was incubated with different concentrations of CTAB (0 - 0.01 %) and stained with Syto^®^ 9 and propidium iodide (PI) to discriminate intact, live from permeabilized, dead bacteria. Images in the Syto^®^ 9 and PI channels were aquired with a 100x objective and appropriate filter sets for excitation and emission of the two dyes (Scale bars: 2 μm). **(B)** *L. monocytogenes* EGD-e/pNZ-P_help_-pHluorin was permeabilized in LMB at different pH containing 0.005% CTAB and, at the indicated pH, excitation spectra (350–490 nm) was aquired measuring relative fluorescence intensity (RFI) at 510 nm emission.

To assess changes in pH_i_, we next sought to measure fluorescence of pHluorin in permeabilized cells of *L. monocytogenes* EGD-e/pNZ-P_help_-pHluorin. Permeabilization of *L. monocytogenes* EGD-e with the quaternary ammonium surfactant cetyltrimethylammonium bromide (CTAB) was efficient without changes in cell morphology at a concentration of 0.005% (w/v; Figure 3A). CTAB-permeabilized *L. monocytogenes* EGD-e/pNZ-P_help_-pHluorin showed a similar ratiometric pH-dependent shift in excitation spectra (Figure 3B) as the crude extracts.

**FIGURE 4:**
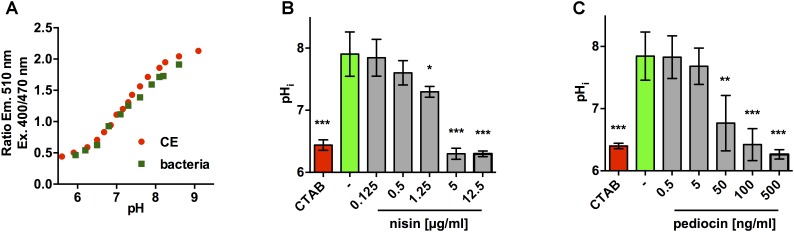
Assay to determine membrane-damaging activity of bacteriocins using the ratiometric fluorescence properties of *L. monocytogenes* EGD-e/pNZ-P_help_-pHluorin. **(A)** Calibration curves of the ratio of fluorescence intensities (emission at 510 nm) after excitation at 400 and 470 nm at different pH for crude extracts (red) and permeabilized bacteria of *L. monocytogenes* EGD-e/pNZ-P_help_-pHluorin (green). **(B,C)** *L. monocytogenes* EGD-e/pNZ-P_help_-pHluorin was incubated in LMB pH 6.5 containing the indicated concentrations of nisin A **(B)** or pediocin PA-1 **(C)** and intracellular pH (pH_i_) was calculated based in the ratio of fluorescence intensities (emission at 510 nm) after excitation at 400 and 470 nm using the calibration curve in **(A)**. CTAB (0.005%) was used as a positive control and untreated bacteria in LMB pH 6.5 without bacteriocin served as negative control (–). Values are mean ± standard deviation of three independent experiments using different cultures of the sensor strain. Statistical analysis was performed by ANOVA with Dunnett’s post-test to calculate *p*-values adjusted for multiple comparisons with untreated bacteria (–) set as control condition (^∗^*p* < 0.05; ^∗∗^*p* < 0.01; ^∗∗∗^*p* < 0.001).

Using the 510 nm emission ratios following excitation at 400 and 470 nm, calibration curves were calculated for pHluorin in crude extracts and permeabilized bacteria that allow conversion of ratio of emission with excitation at 400 and 470 nm to pH values (Figure 4A). Based on the calibrations curves, we reasoned that mild acidic buffer (pH 6.5) would be suitable and sufficient to establish an assay to monitor changes in pH_i_ following membrane damage. To demonstrate functionality of this assay, *L. monocytogenes* EGD-e/pNZ-P_help_-pHluorin was resuspended in buffer at pH 6.5, exposed a range of concentrations of nisin A, a bacteriocin with known membrane-damaging activity, and fluorescence at 510 nm was measured following excitation at 400 and 470 nm. The obtained ratios were then used to calculate pH_i_ of bacteria under the tested conditions (Figure 4B). Untreated bacteria had a pH_i_ of 7.8–7.9 and permeabilization with 0.005% (w/v) CTAB resulted in change of pH_i_ to pH_o_ of the buffer (pH = 6.4–6.5) indicating a complete loss of membrane integrity. Nisin A had a dose-dependent effect on pH_i_. No changes in pH_i_ were observed in the presence of 5 and 20 μg/ml. By contrast, 200 and 500 μg/ml of nisin A resulted in complete drop of pH_i_ to 6.4–6.5 (i.e., pH_i_ = pH_o_) and 50 μg/ml resulted in a significant, yet incomplete drop in pH_i_. Similar results, however, at 25–50 times lower concentrations, were observed with pediocin PA-1 (Figure 4C). At 0.5 and 5 ng/ml, no effect on pH_i_ was observed, at 100 and 500 ng/ml pH_i_ was reduced to pH_o_, and at 50 ng/ml a significant yet partial reduction in pH_i_ was observed.

**FIGURE 5:**
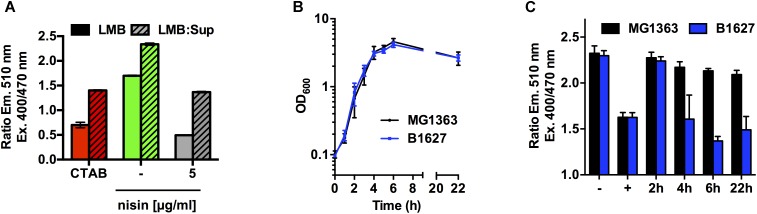
Assay to determine membrane-damaging activity in supernatants of bacteriocin producing bacteria using *L. monocytogenes* EGD-e/pNZ-P_help_-pHluorin. **(A)** *L. monocytogenes* EGD-e/pNZ-P_help_-pHluorin was incubated at pH 6.5 in either LMB (solid bars) or a 1:1 mix of LMB with supernatant of *L. lactis* MG1363 (LMB:Sup; hatched bars) containing 5 μg/ml nisin. CTAB (0.005%) was used as a positive control (+) and untreated bacteria in either LMB or LMB:Sup without bacteriocin served as negative control (-). **(B)** OD_600_ of *L. lactis* MG1363 (black) or B1627 (blue) grown in GM17 medium. **(C)** Supernatants were collected for the cultures shown in **(B)** at the indicated time points during growth and incubated with *L. monocytogenes* EGD-e/pNZ-P_help_-pHluorin (1:1 mix of supernatant with sensor strain in LMB). Values in **(A)** and **(C)** are ratios of fluorescence intensities (emission at 510 nm) after excitation at 400 and 470 nm and are mean ± standard deviation of two **(A)** or four **(C)** independent experiments using different supernatants and different cultures of the sensor strain.

As a next step, we wanted to test the possibility to detect membrane-damaging substances with the sensor strain not only in a “clean” buffer system but also in more complex mixtures such as media used for cultivation of e.g., bacteriocin-producing strains. When *L. monocytogenes* EGD-e/pNZ-P_help_-pHluorin was incubated in LMB pH 6.5 mixed in a 1:1 ratio with spent culture supernatants of *L. lactis* MG1363, which does not produce a bacteriocin, spiked with 200 μg/ml of nisin, a similar drop of pH_i_ to the pH_o_ as observed with LMB alone (Figure 5A). The ratios of emission with excitation at 400 and 470 nm were considerably higher in the mix of buffer with supernatant than in plain buffer. However, a comparison of emission ratios (at 510 nm) after excitation at 400 and 470 nm was considered sufficient to simply assess the effect of membrane-damaging compounds. In order to convert emission ratios to intracellular pH, an individual calibration curves need to be calculated for a specific condition or buffer system individually based on excitation spectra aquired with *L. monocytogenes* EGD-e/pNZ-P_help_-pHluorin at different pH.

We further explored the potential of the sensor strain to detect active bacteriocins in the supernatants of a nisin producer strain. The two *L. lactis* strains MG1363 (non-producing type strain) and B1627 (nisin Z-producing isolate) showed comparable growth in GM17 medium and no difference in final optical density was observed (Figure 5B). Supernatants of the non-producing type strain MG1363 had no effects on the ratio of emission with excitation at 400 and 470 nm, and thus pH_i_ of *L. monocytogenes* EGD-e/pNZ-P_help_-pHluorin was not affected (Figure 5C). By contrast, supernatants of the nisin Z producer *L. lactis* B1627 reduced the ratio of emission with excitation at 400 and 470 nm in a time-dependent manner (Figure 5C). Supernatants collected after 2 h of growth (i.e., early exponential growth phase and low biomass) did not change pH_i_. By contrast, supernatant collected after 4, 6, and 22 h (i.e., mid-exponential to stationary growth phase and high biomass) reduced pH_i_ to a similar extent as the positive control CTAB indicating a concentration of active nisin Z sufficient for complete disruption of membrane integrity.

**FIGURE 6:**
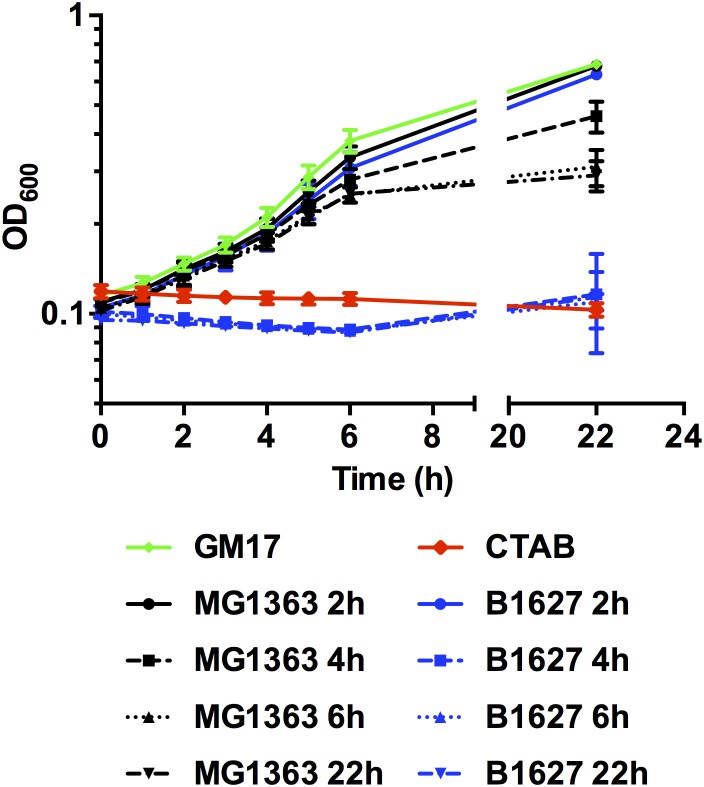
Growth of *L. monocytogenes* EGD-e/pNZ-P_help_-pHluorin in the presence of *L. lactis* supernatants. *L. monocytogenes* EGD-e/pNZ-P_help_-pHluorin was incubated in BHI mixed (1:1 ratio) with supernatants of *L. lactis* MG1363 (black) or B1627 (blue) collected at the indicated timepoints of growth in GM17 (i.e., the supernatants tested in Figure 5). GM17 with CTAB (0.005%) was used as a positive control (red) and sterile GM17 without any supplements served as negative control (green). Growth of *L. monocytogenes* EGD-e/pNZ-P_help_-pHluorin was monitored by measuring OD_600_. Values are mean ± standard deviation of two independent experiments each measured in technical duplicates using different supernatants and different cultures of the sensor strain.

Finally, the capacity of the supernatants containing membrane-damaging levels of nisin to inhibit growth of a target organism was tested. In line with the observations on pH_i_, supernatants of the nisin Z producer *L. lactis* B1627 collected after 4, 6, and 22h of growth efficiently inhibited growth of *L. monocytogenes* EGD-e/pNZ-P_help_-pHluorin (Figure 6) to a similar extent as CTAB. By contrast no inhibition of growth was observed, with plain GM17 broth or supernatants collected from either of the two *L. lactis* strains after 2 h of growth, i.e., supernatants that did not change pH_i_. A slight reduction in final OD of *L. monocytogenes* EGD-e/pNZ-P_help_-pHluorin was observed with supernatants of the non-producer *L. lactis* MG1363 collected at later timepoints, which may be due to a reduced nutrient content and/or low pH in the mix of BHI with *L. lactis* supernatants of the late exponential to stationary growth phase.

## Discussion

In previous studies, pHluorin was used to investigate membrane homoeostasis of *Escherichia coli* and *Bacillus subtilis* following acid stress (Martinez et al., 2012), proton leakage through the MotA/B proton channel of the flagellar apparatus of *Salmonella enterica* (Morimoto et al., 2010), or the effect of potassium uptake on metabolism of *Staphylococcus aureus* (Gries et al., 2016). Moreover, pHluorin has been successfully used in *B. subtilis* both on population and single cell level to study antibacterial effects of weak organic acids to assess their potential as food preservation strategies (Ter Beek et al., 2015; Pandey et al., 2016). In the present study, we investigated the potential of pHluorin to assess membrane-damaging activity of bacteriocins in *L. monocytogenes*.

Following successful cloning and expression of pHluorin in *L. monocytogenes* EGD-e/pNZ-P_help_-pHluorin (Figure 1) we analyzed the fluorescence properties of the protein in crude extracts and permeabilized cells at different pH (Figures 2, 3). In line with the original study (Miesenböck et al., 1998), pHluorin showed decreased emission at the 400 nm excitation peak with decreasing pH. However, we did not observe a concomitant increase in emission at the 470 nm excitation peak as described by Miesenböck et al. (1998). This may be due to the fact that excitation spectra were obtained in crude extracts or permeabilized bacteria in LMB whereas the spectra obtained by Miesenböck et al. (1998) were aquired with purified protein in a well-defined buffer system (50 mM sodium cacodylate, 50 mM sodium acetate, 100 mM NaCl, 1 mM CaCl_2_, 1 mM MgCl_2_). Despite the absence of an increase in fluorescence intensity following excitation at 470 nm, the ratiometric response of the protein were almost identical in crude extracts and permeabilized bacteria (Figure 4A). Based on the calibration curve of permeabilized bacteria, LMB with a pH of 6.5 was selected to assay bacteria for membrane-damaging activity of nisin A and pediocin PA-1. Nisin A led to complete disruption of membrane integrity of *L. monocytogenes* EGD-e/pNZ-P_help_-pHluorin at a concentration between 1.25 and 5 μg/ml. This is in line a recent study reporting a minimal inhibitory concentration of 2 μg/ml for *L. monocytogenes* using purified nisin A in a growth-dependent assay (Zhou et al., 2015). For pediocin PA-1, complete decrease of pHi to pH_o_ was observed at a concentration between 50 and 100 ng/ml (i.e., 1.1–2.2 nM), which is also in the range of the MIC for *L. monocytogenes* recently determined using chemically synthesized pediocin PA-1 (6.8 nM; Bédard et al., 2018).

Interestingly, pH_i_ was significantly but not completely decreased to pH_o_ at intermediate concentrations of nisin A (1.25 μg/ml) or pediocin PA-1 (50 ng/ml). This may indicate that there is incomplete membrane damage with disruption of the membrane potential and (slow) leakage of protons across the membrane but this damage is not (yet) extensive enough to cause immediate and complete disruption of pH homeostasis. Thus, bacteria may still be able to counteract pH stress and to pump protons. However, it is also possible that the signal (i.e., the change in excitation ratio) is obtained from a mixed population of bacteria, in which some cells have perfectly intact membranes (and an excitation ratio of intact cells) while others have a completely disrupted membrane (with an associated excitation ratio of pH_i_ = pH_o_). This has to be analyzed in further experiments with e.g., extended incubation times and single cell analysis by fluorescence microscopy or flow cytometry with appropriate instrument setups for the excitation/emission properties of pHluorin.

Nisin and other lantibiotic bacteriocins were shown to have distinct modes of action that are mediated by binding to lipid II (Wiedemann et al., 2001; Breukink and de Kruijff, 2006). On the one hand, lipid II is an essential carrier molecule for cell wall precursors. Binding of nisin to lipid II inhibits cell wall synthesis and thus growth. On the other hand, complexes of lipid II and nisin form pores, which have a nisin:lipid II stoichiometry of 8:4 in unilamellar lipid vesicle (Hasper et al., 2004). Measurements of pH_i_ using pHluorin only assesses membrane damage and thus are a readout for the pore-forming activity of bacteriocins (and other compounds). Compounds that act only on the Ψ potential of the proton motif force or inhibit growth by other mechanisms are not detected by the sensor strain. However, when coupled with a readout to assess growth, e.g., by classical OD_600_ measurement, they may provide further information, whether a compound acts by growth inhibition or membrane disruption.

In conclusion, our results demonstrate successful and fast analyses of the activity of membrane targeting bacteriocins on *L. monocytogenes* by measuring pH_i_ using pHluorin. Thus, expression of pHluorin is a convenient approach to identify and test novel antimicrobial compounds active against *L. monocytogenes* and other (human) pathogens. Additionally, *L. monocytogenes* EGD-e/pNZ-P_help_-pHluorin and derivatives in combination with fluorescence ratio imaging microscopy (Martinez et al., 2012; Pandey et al., 2016) can be exploited for infection research to study the mechanisms of pH-homeostasis of *L. monocytogenes* e.g., in highly acidic phagosomes/phagolysosomes macrophages and other host cells.

## Author Contributions

CR and GS conceived the study. PC, LS, and DW carried out the experiments. DD provided the reagents. PC, NB, DD, CR, and GS analyzed data. PC, DW, CR, and GS drafted the manuscript. All authors contributed to preparing the final version of the manuscript and read and approved the final manuscript.

## Conflict of Interest Statement

The authors declare that the research was conducted in the absence of any commercial or financial relationships that could be construed as a potential conflict of interest.
